# Prophylactic Drain Placement in Childhood Perforated Appendicitis: Does Spillage Matter?

**DOI:** 10.3389/fped.2020.588109

**Published:** 2020-10-09

**Authors:** Yannick Michael Schmidt, Danielle Wendling-Keim, Dietrich von Schweinitz, Jochen Hubertus, Michael Berger

**Affiliations:** Department of Pediatric Surgery, Research Laboratories, Dr. von Hauner Children's Hospital, Ludwig Maximilian University of Munich, Munich, Germany

**Keywords:** perforated appendicitis, prophylactic drain placement, appendecitis, appendectomy, appendicitis complications

## Abstract

**Background:** Prophylactic abdominal drains for perforated appendicitis in children have generally been regarded as obsolete because several studies showed inferior results for drain placement in the past. Despite these results, prophylactic abdominal drains for perforated appendicitis remain omnipresent in pediatric surgery especially when gross spillage is observed at the time of appendectomy. Here, we hypothesize that even if accounting for gross intra-abdominal spillage, prophylactic drain placement for perforated appendicitis in children is not beneficial.

**Patients and Methods:** The charts of all children (<18 years) who underwent an appendectomy at our institution from July 2013 to March 2020 were analyzed. The data from 65 patients who presented with perforated appendicitis were included. Patients were grouped according to the amount of intraoperative spillage. Demographics, laboratory data, operative findings, and postoperative outcomes were analyzed.

**Results:** Of all patients, 34 were male, and 31 were female, with a mean age of 10.5 ± 3.7 years. There were no statistically significant differences between the groups for age and sex (*p* = 0.6985 and *p* = 0.6222, respectively). Prophylactic drains were placed according to the surgeon's preference in 32 children. There were no statistically significant differences between the groups in the rate of intra-abdominal abscess formation, wound infection, and bowel obstruction, regardless of the amount of spillage encountered during an appendectomy. However, independently of the amount of spillage, the length of hospital stay was longer in the children in which a drain had been placed (*p* = 0.0041).

**Conclusion:** In our cohort, we could not find a benefit from drain placement even in case of gross spillage at the time of appendectomy. Rather, drain placement was associated with an increase in length of hospital stay.

## Introduction

Appendicitis is common in children. Especially younger children under the age of 5 years often present with a perforated appendix (1). Perforation, in all age groups, is associated with postoperative complications such as intra-abdominal abscess formation, which has been reported to have an incidence as high as 18% in comparison with 1–2% after non-perforated appendicitis (2, 3). To date, prophylactic placement of a peritoneal drain during appendectomy is not regarded as beneficial to prevent postoperative abscess formation (4–6). Although some authors still describe a reduction of complications due to the usage of intraperitoneal drains (7), most articles nowadays state that placing abdominal drainage does not prevent postoperative abscess formation and may even lead to an increase of postoperative complications (8, 9). Despite this evidence, some surgeons opt to place prophylactic drains, especially in cases of perforated appendicitis, in which the gross amount of intra-abdominal spillage is noted. Consequently, despite existing evidence, prophylactic drain placement in perforated appendicitis remains omnipresent in pediatric surgery.

To our knowledge, in no study has the intra-abdominal finding and the extent of the inflammation at the time of appendectomy been included (7–9). Without such distinction, however, results may be skewed by selection bias. Perforated appendicitis is not homogenous in its presentation. Rather, it can involve a spectrum of symptoms ranging from covered local perforation without spillage that mimics simple appendicitis to gross spillage of pus into the abdominal cavity with subsequent fulminant septic shock from peritonitis. Therefore, oftentimes patients receive a drain because they are sicker than others, making a comparison of these patients difficult. It has been criticized that this selection bias may be the culprit for some of the inferior results that have recently emerged for patients who were treated with abdominal drains in perforated appendicitis.

Therefore, in this study, we sought to analyze the outcome of children treated in our hospital for perforated appendicitis and taking into account the severity of inflammation, as indicated by both preoperative laboratory values as well as the intraoperative amount of pus in the abdominal cavity at the time of appendectomy. We hypothesize that, even if controlled for the amount of intra-abdominal spillage, placing a prophylactic abdominal drain is not beneficial.

## Patients and Methods

The charts of all patients who underwent an appendectomy at our institution from July 2013 to March 2020 were reviewed. Initially, 68 patients were found in the records. To generate a heterogeneous patient population for further analysis, three patients were excluded (two with severe neurological dysfunction and one with inflammatory bowel disease, see Supplementary Table 1). The data from the remaining 65 patients with an age range from 2 to 17 years who presented with perforated appendicitis were analyzed. The diagnosis was confirmed by reviewing the histology of all corresponding cases. Unless the pathologist documented perforation, patients were excluded. Prophylactic drains were placed according to the surgeon's preference in 32 children. Thirty-three patients did not receive a drain. The study population was grouped according to the amount of intra-abdominal spillage (no spillage, minimal spillage, and gross spillage). Demographics, laboratory data, operative findings, and postoperative outcomes were analyzed.

All patients with perforated appendicitis received prophylactic postoperative antibiotic treatment with cefuroxime and metronidazole, which was continued depending on their intraoperative findings and their individual course. The attending surgeon, on individual bases, decided whether to place a peritoneal drain. Drains were eliminated after 24–72 h according to the amount and consistency of the drained fluid.

Statistical analysis was carried out using Graphpad Prism version 8. The significance level was set at *p* values ≤ 0.05.

## Results

We included 65 patients aged 2 to 17 years who underwent appendectomy for perforated appendicitis during the time period from July 2013 to March 2020. There were 31 female and 34 male patients. The mean age was 10.5 ± 3.7 years. The two groups did not differ significantly regarding their age (*p* = 0.6985) or sex (Table 1).

**Table 1:** Baseline characteristics of patients with perforated appendicitis.

	**All *n* = 65**	**No drain *n* = 33**	**Drain *n* = 32**	***p*-value**
Age [y]	10.53	10.70	10.34	0.6985^a^
Gender [m/f]	34/31	16/17	18/14	0.6222^b^
WBC [G/l]	16.65	16.62	16.68	0.9622^a^
CRP [mg/dl]	8.45	7.50	9.40	0.2000^c^
Type of operation (%)				
Laparoscopic	57	28 (49.12)	29 (50.88)	
Open	6	5 (83.33)	1 (16.66)	
Conversion	2	0 (0)	2 (100)	

a *Unpaired t-test*.

b *Fisher's exact test*.

c *Mann-Whitney U test*.

**Figure 1:**
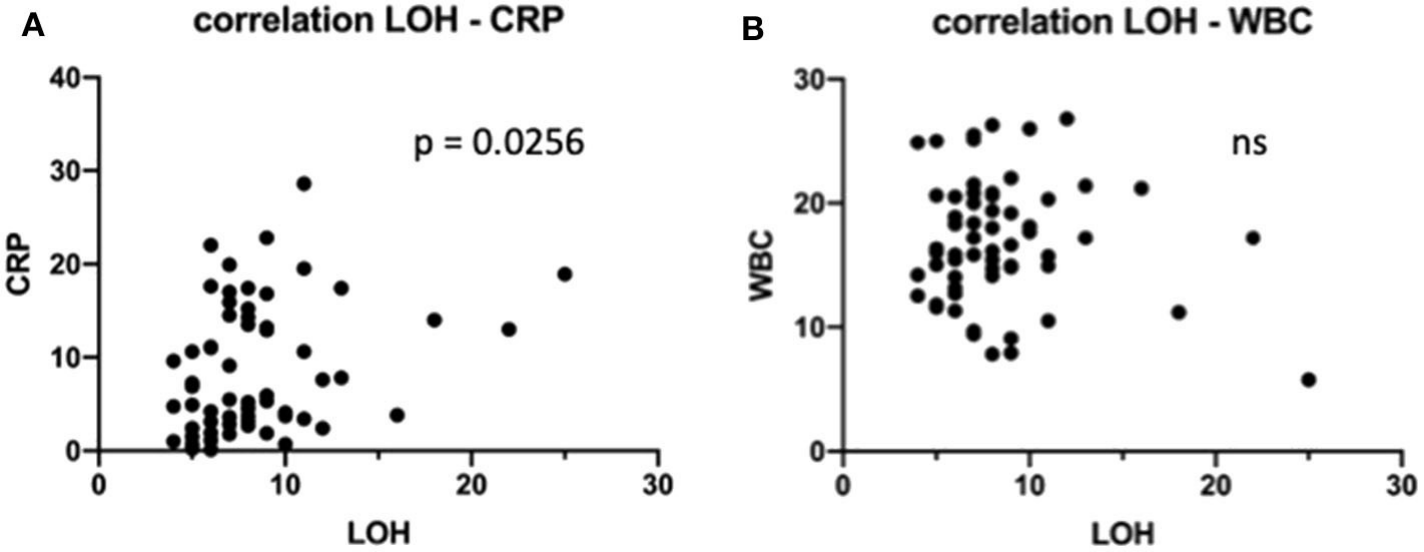
Impact of preoperative laboratory on length of the hospital stay (LOH). **(A)** C-reactive protein levels were correlated to LOH in all children. **(B)** White blood cell levels were correlated to LOH in all children. ns, not significant.

Of these 65 children, in 32 patients (49.2%), a peritoneal drain was prophylactically inserted, whereas the other 33 patients did not receive a peritoneal drain. For further analysis of our patient population and for comparison between groups, we considered preoperative inflammation markers C-reactive protein (CRP) and white blood cell (WBC) count. When grouping patients according to drain placement and to analyze for CRP and WBC, we found no statistical difference between the drain and non-drain groups (*p* = 0.2 and *p* = 0.9622, respectively; Table 1). Preoperative CRP or WBC did not correlate with abscess formation or any other outcome marker measured. Interestingly, however, there is one notable exception, which is a correlation of preoperative CRP with the length of the hospital stay (LOH) (*r* = 0.2790; *p* = 0.0256; Figure 1A). There was no correlation between the WBC and the LOH (*p* = 0.5795; Figure 1B).

**Figure 2:**
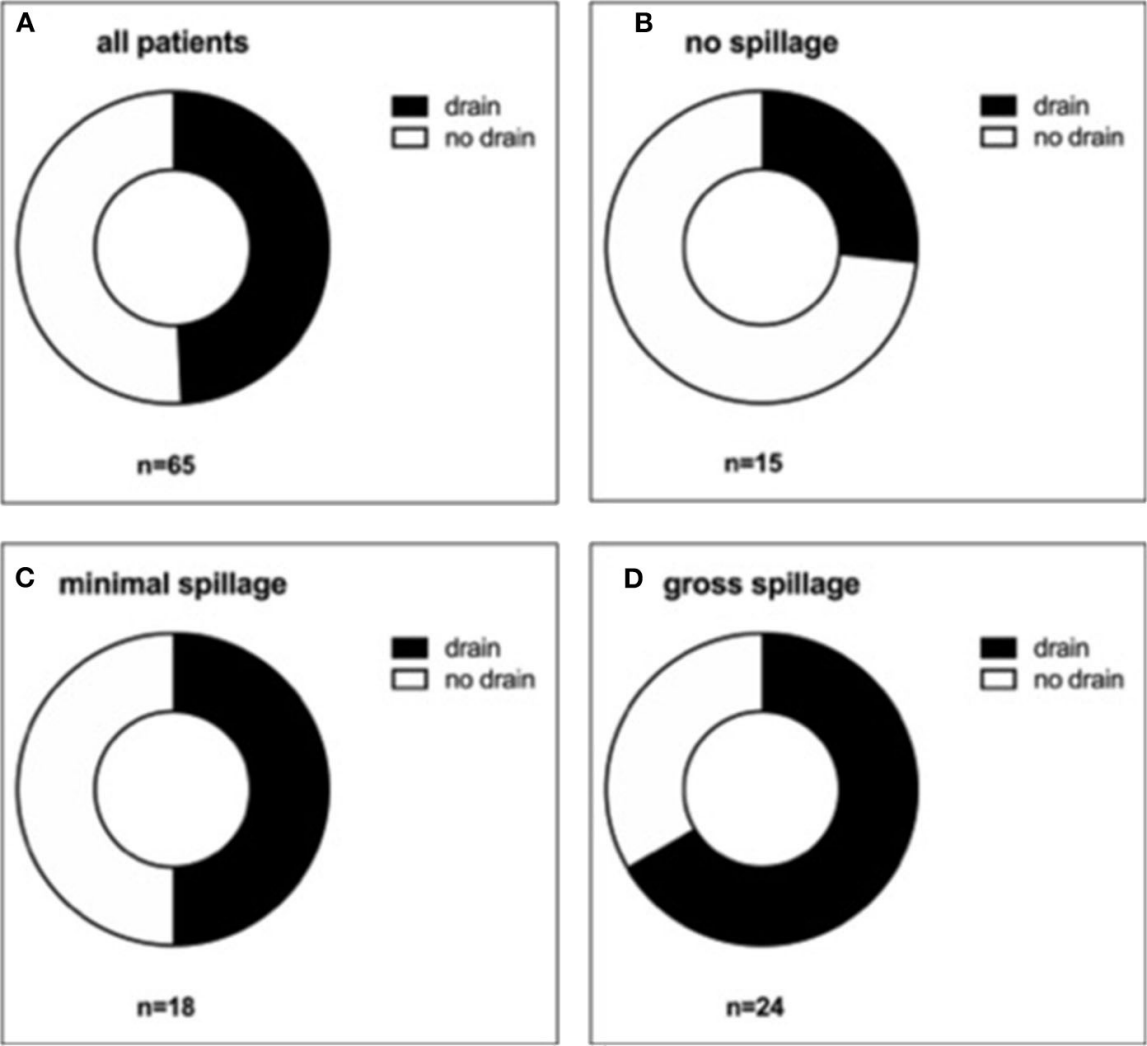
Distribution of drain placement according to the amount of intra-abdominal spillage. All patients **(A)**, those with no spillage **(B)**, with minimal spillage **(C)**, and gross spillage **(D)** were analyzed regarding drain placement.

Given the limitations of using laboratory data alone to assess how fare these children's inflammatory process was advanced at the time of appendectomy, we further grouped all patients according to their intraoperative findings. In 15 patients (23.1%), it was documented that no spillage of pus had occurred. In 18 patients (27.7%), minimal spillage and, in 24 patients (36.9%), gross spillage were explicitly documented in the OR report (Figure 2). In eight children (12.3%), neither the amount nor presence of spillage was commented on, or the documentation was conflicting. Of the later eight children, three had a drain placed.

**Figure 3:**
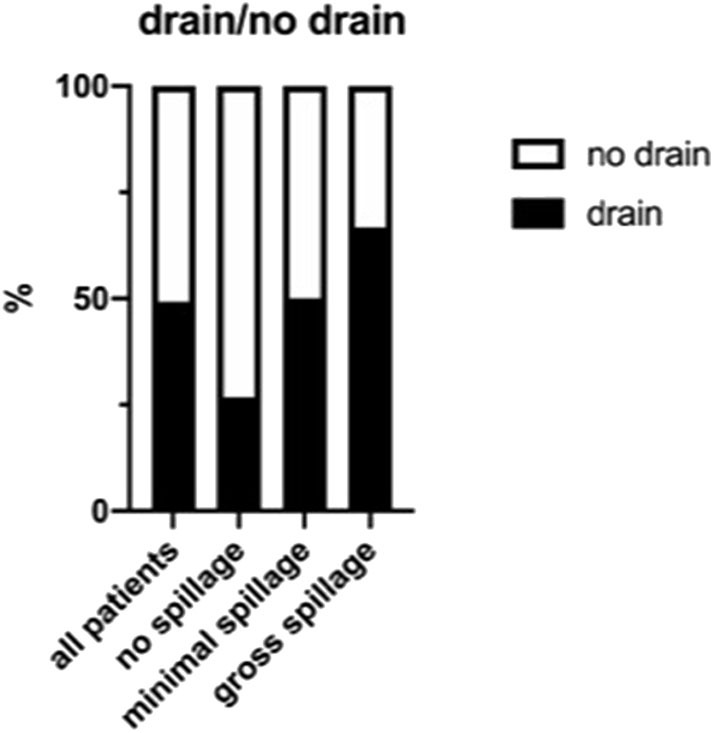
Percentage of drain placement according to the amount of intra-abdominal spillage.

A subgroup analysis according to the amount of spillage showed that compared with the group with no spillage, the percentage of patients receiving a drain was higher in the group for minimal spillage (26.7 vs. 50.0%, respectively) and highest in the group with gross spillage (66.7%) (Figure 3). Therefore, as one can expect, the more severe the appendicitis appeared to the surgeon intraoperatively, the more likely he or she was to place a drain.

**Figure 4:**
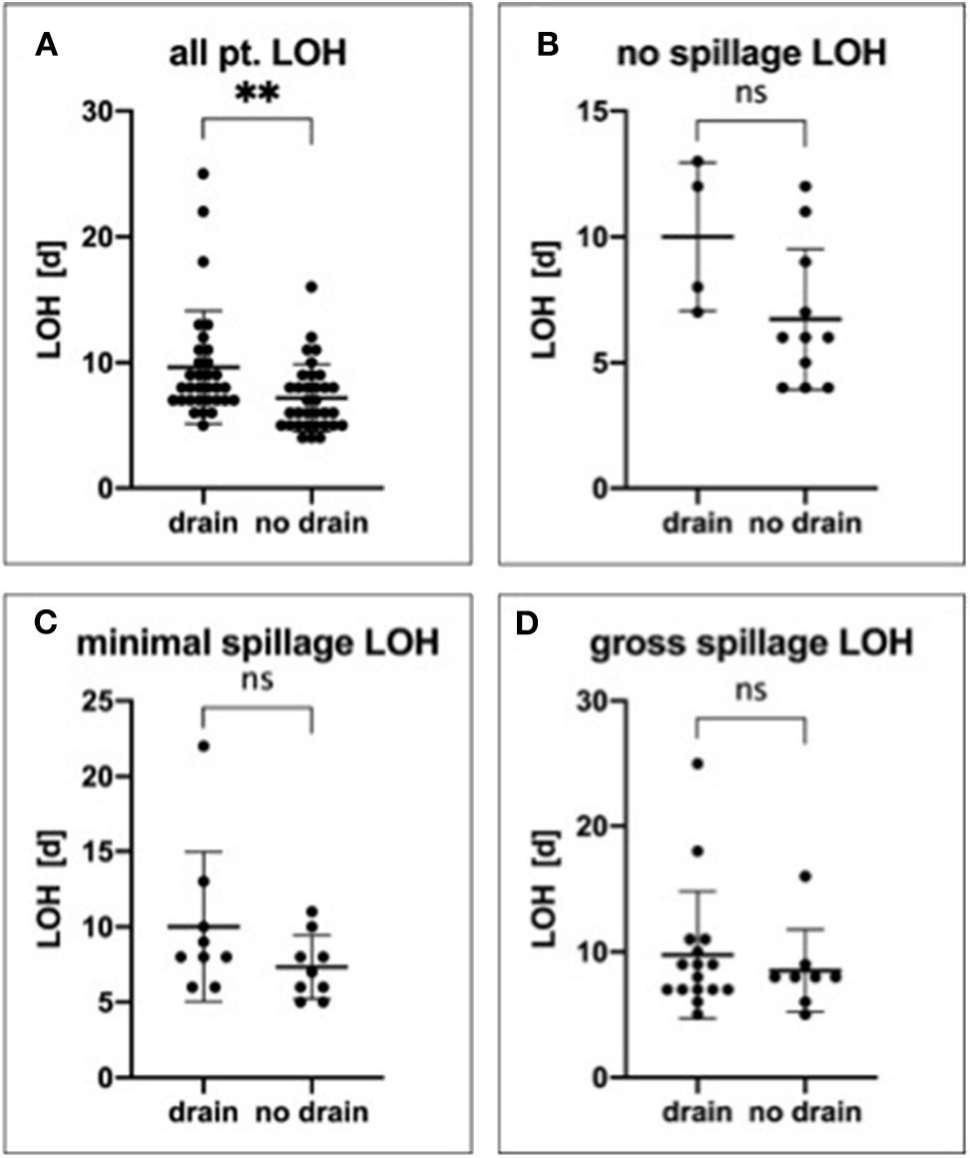
Impact of drain placement on length of the hospital stay. All patients **(A)**, those with no spillage **(B)**, with minimal spillage **(C)**, and gross spillage **(D)** were analyzed regarding LOH and drain placement. **P ≤ 0.01; ns, not significant.

Given that our results so far had shown a statistically significant correlation between preoperative CRP and LOH, we further analyzed LOH as a surrogate parameter. Notably, we found that, when regarding all patients, the patient group who received a drain had a significantly prolonged hospital stay (*p* = 0.0041; Figure 4) in comparison with the group without a drain. However, given our observation that there was a correlation between CRP and LOH, it is possible that the observed correlation between drain placement and LOH stems not from the drain placement itself but from the fact that the involved patients were sicker to begin with. This holds true potentially even in the case that, as was observed in our cohort, neither WBC nor CRP correlated with drain placement. In this same sense, neither did WBC correlate with LOH (*p* = 0.5795).

Therefore, to minimize selection bias, we performed further subgroup analysis for all outcome markers, including LOH, according to the amount of spillage. In detail, for all groups combined, the mean LOHs were 8.0 (5–25) days with a drain and 6.0 (4–16) days without a drain (*p* = 0.0041). In the no spillage group, the mean LOHs were 10.0 (7–13) days with a drain and 6.7 (4–12) days without a drain (*p* = 0.0693). Further, in the minimal spillage group, the mean LOHs were 10.0 (6–22) days with a drain and 7.33 (5–11) days in the patients without a drain (*p* = 0.1585), whereas in the gross spillage group, the LOHs were 8.5 (5–25) days with a drain and 8.0 (6–16) days in the patients without a drain (*p* = 0.6155). Therefore, in all subgroups analyzed, the LOH was shorter in patients without a drain, although this trend did not reach statistical significance in any of the subgroups (Figure 4).

**Figure 5:**
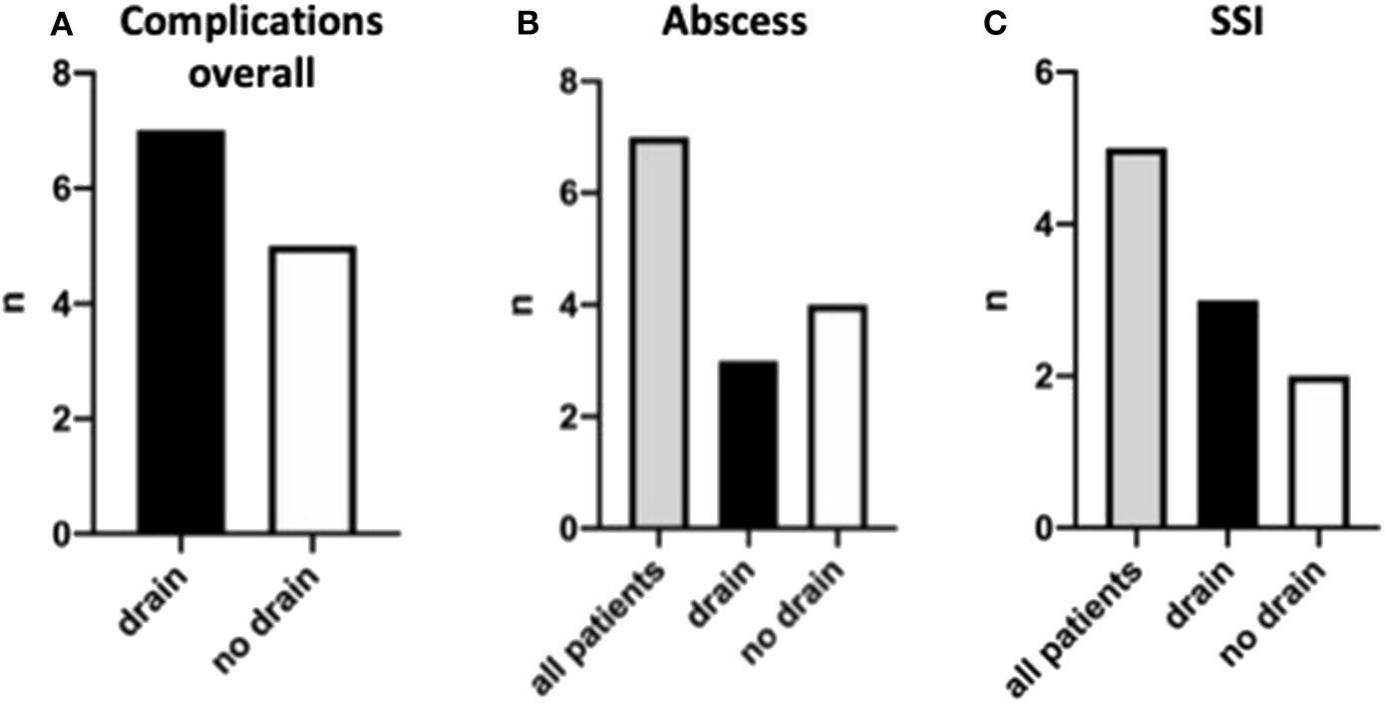
Post-surgery complications and their distribution. Complications are shown overall **(A)**, for intra-abdominal abscess formation **(B)**, and surgical site infections **(C)**.

In the last step, we analyzed our patient cohort for postsurgical complications, including intra-abdominal abscess formation, surgical site infection, need for readmission, and redo-surgery. Overall, we observed 15 complications in 12 children (18.4%, Table 2). Of these 12 children, 7 (58.3%) had a drain placed at the time of appendectomy, and 5 (41.7%) did not (Figure 5A). In detail, seven children (10.8%) had intra-abdominal abscess formation. Of this group, three had a drain placed at the time of appendectomy, and four had no drain (Figure 5B). Three of these seven children needed to be reoperated. Of the 65 children, 5 (7.7%) suffered from surgical site infection, and 4 of these children needed to be reoperated. Three of the five children initially had a drain placed (Figure 5C). Apart from these complications, one child developed peritonitis postoperatively and had to be readmitted for further antibiotic therapy. We also observed a small bowel obstruction in one child requiring readmission and surgical exploration as well as one child who had originally presented with gross spillage and had drains placed, and who later developed a pleural effusion in addition to a surgical site infection.

**Table 2:** Postoperative complications.

**Complication**	**Operative technique**	**Drain**	**Gender**	**Age [y]**	**Re-surgery**	**Re-admission**
Umbilical abscess	Laparoscopic	No	Female	4.89	Yes	Yes
Intraabdominal + wound abscess	Laparoscopic	Yes	Female	8.64	Yes	Yes
Abdominal wall abscess	Open surgery	Yes	Male	4.33	Yes	No
Serom prevesical	Laparoscopic	No	Female	12.39	Yes	No
Intraabdominal abscess	Laparoscopic	Yes	Male	10.45	Yes	No
Intraabdominal retained fluids	Laparoscopic	Yes	Male	9.06	No	No
Intraabdominal retained fluids + small wound infection	Laparoscopic	No	Male	15.94	No	No
Intraabdominal abscess	Laparoscopic	No	Male	5.53	No	No
Obstructive ileus due to adhesions	Laparoscopic	Yes	Male	11.62	Yes	Yes
Intraabdominal retained fluids	Laparoscopic	No	Female	11.02	No	No
Peritonitis	Laparoscopic	Yes	Female	10.77	No	Yes
Abdominal wall abscess + pleural effusion	Conversion	Yes	Female	15.92	Yes	No

## Discussion

Although perforated appendicitis is a common cause of acute abdomen and abdominal surgery in children and adults alike (10), there is still controversy about whether a prophylactic peritoneal drain is necessary and whether it leads to a better outcome with reduced postoperative complications. In some studies, the prophylactic placement of a peritoneal drain is described as beneficial, whereas others question its benefit or found that it leads to more complications (4, 6, 7, 11, 12). To our knowledge, no study so far has assessed whether the amount of intra-abdominal spillage impacted the outcome in the setting of whether or not a drain was placed.

Therefore, we sought to address this question. We selected patients aged 2 to 17 years who underwent appendectomy for perforated appendicitis at our center from July 2013 to March 2020. The outcome of those children who received a prophylactic drain at the time of appendectomy was compared in retrospect with those children who did not. Further, not only did we consider preoperative CRP and WBC but also differentiated three groups according to the amount of spillage of pus (no spillage, minimal spillage, and gross spillage). By analyzing the LOH as our primary outcome measure under consideration of the severity of appendicitis, we sought to minimize a selection bias that may be attributable to mistakenly comparing patients with different grades of peritoneal inflammation. This approach has not been applied in the context of the mentioned research question before.

Interestingly, in our cohort, we found that the LOH was significantly increased in patients with a drain when considering all patients. We also found a trend of the LOH being lower in patients without peritoneal drainage when differentiating the patients into the different spillage groups. Although this subgroup analysis lacked statistical significance, the absolute numbers were consistent in all subgroups. Likely, this finding is influenced by the small size of the subgroups, which reduced statistical power.

The described results regarding the LOH were further examined in the light of the occurrence of postoperative complications, such as postoperative intra-abdominal abscess formation, surgical site infection, readmission, and redo-surgery. These complications were rare in both groups; hence, no differences were detected in the complication rate between the drain group and the group without a drain.

Our findings are in accordance with other reports. Aneiros Castro et al. (8) reported on 192 children who underwent laparoscopic appendectomy for perforated appendectomy and found no benefit for placing a prophylactic drain. Rather, in their report, increased requirements of antibiotic and analgesic medication, fasting time, operative time, and LOH were observed when placing a drain. In 2014, similar findings had been reported by Song et al. (9). Their analysis of 342 patients with perforated appendicitis showed that insertion of a peritoneal drain at the time of appendectomy was associated with higher complication rates, including the formation of intra-abdominal abscesses. Further, similar to our results, LOH was increased for children in whom a drain had been placed. In accordance with these results, Akkoyun and Tuna (5), reported on a total 234 children with perforated appendicitis, comparing drainage and irrigation versus no drainage and no irrigation. They found no statistically significant difference in postoperative infectious complications between both groups. Again, similar to Song et al., Castro et al., Akkoyun et al., and our results, the length of postoperative hospital stay was significantly longer in the drain group. Also, the operative time was significantly longer. Similar findings were reported in 2007 by Narci et al. (6). Analyzing a total of 226 children with perforated appendicitis, they found significantly more complications, including intra-abdominal abscess formation and wound infection if a drain was placed. Additionally, they found that the postoperative hospitalization period and the durations of antibiotic use, nasogastric tube usage, time to oral feeding, and time to normalization of the body temperatures were significantly longer if a drain was used. All these findings are in accordance with the findings described in our study. Unlike in our report, however, in none of these studies, a distinction was made between the amounts of spillage encountered at the time of appendectomy, potentially creating selection bias of the study material.

Our results stand in contrast to findings described by Beek et al. who described 199 patients with perforated appendicitis who underwent appendectomy (7). According to their report from 2015, prophylactic peritoneal drain placement leads to a reduced rate of complications, including intra-abdominal abscess formation and wound infection. However, different from our report, this analysis was performed exclusively in adult patients. A comparison with our pediatric patient population is, therefore, difficult, if not impossible.

Our study has several limitations. First, we used a retrospective design, which did not allow for standardized decision-making when to insert a drain. Rather, the decision of whether to place a drain was made by the individual surgeon. However, when we differentiated our results according to the amount of spillage that was recorded in the operating report, we found the judgment of the various surgeons to be consistent throughout the study in that the amount of spillage found at the time of appendectomy clearly correlated with the likelihood to place a drain. If anything, our findings support the importance of distinguishing patients with more severe disease from others with less severe disease to avoid selection bias.

On the first approach, a further limitation of our study seems to be that the number of patients in our subgroup analysis was too small to generate enough statistical power to generate statistically significant differences. This being so, it is important to note that the intention of our study is not to prove that placing a prophylactic peritoneal drain at the time of appendectomy for perforated appendicitis is inferior or superior to not placing a drain. Rather, our study intends to analyze the clinical relevance of placing or not placing a drain. Where there no difference in outcome detected between the two groups in reasonable sample size, such as is the case in the study presented here, from a clinical perspective, this finding would inevitably favor a decision not to place a drain in the described context. Therefore, although we cannot claim that potentially a statistically significant difference exists even within our subgroup analysis, and which could be detected by increasing the sample size, we can safely claim that a statistically significant difference picked up that way would likely lack clinical relevance.

In summary, in our cohort of children operated on for perforated appendicitis, using a prophylactic peritoneal drain did not seem to result in a beneficial outcome. Rather, we observed a longer LOH for these children, even when controlled for by the intraoperative amount of spillage.

## Data Availability Statement

All datasets generated for this study are included in the article/Supplementary Material.

## Ethics Statement

This article does not contain any studies with animals performed by any of the authors. All procedures performed in studies involving human participants were in accordance with the ethical standards of the institutional and/or national research committee and with the 1964 Helsinki declaration and its later amendments or comparable ethical standards. Informed consent was obtained from all individual participants included in the study.

## Author Contributions

MB conceptualized the research project and its details, gathered and analyzed data, and wrote the manuscript. YS analyzed data and wrote the manuscript. YS, DW-K, and JH analyzed data. DS reviewed data and the manuscript. All authors contributed to the article and approved the submitted version.

## Conflict of Interest

The authors declare that the research was conducted in the absence of any commercial or financial relationships that could be construed as a potential conflict of interest.

## References

[1] Newman K Ponsky T Kittle K Dyk L Throop C Gieseker K. Appendicitis 2000: variability in practice, outcomes, and resource utilization at thirty pediatric hospitals. J Pediatr Surg. (2003) 38:372–9. 10.1053/jpsu.2003.50111 12632352

[2] St Peter SD Sharp SW Holcomb GW Ostlie DJ. An evidence-based definition for perforated appendicitis derived from a prospective randomized trial. J Pediatr Surg. (2008) 43:2242–5. 10.1016/j.jpedsurg.2008.08.051 19040944

[3] St Peter SD Tsao K Spilde TL Holcomb GW Sharp SW Murphy JP. Single daily dosing ceftriaxone and metronidazole vs standard triple antibiotic regimen for perforated appendicitis in children: a prospective randomized trial. J Pediatr Surg. (2008) 43:981–5. 10.1016/j.jpedsurg.2008.02.018 18558169 PMC3082440

[4] Gasior AC Marty Knott E Ostlie DJ St Peter SD To drain or not to drain: an analysis of abscess drains in the treatment of appendicitis with abscess. Pediatr Surg Int. (2013) 29:455–8. 10.1007/s00383-013-3262-3 23344151

[5] Akkoyun I Tuna AT. Advantages of abandoning abdominal cavity irrigation and drainage in operations performed on children with perforated appendicitis. J Pediatr Surg. (2012) 47:1886–90. 10.1016/j.jpedsurg.2012.03.049 23084202

[6] Narci A Karaman I Karaman A Erdogan D Cavusoglu YH Aslan MK. Is peritoneal drainage necessary in childhood perforated appendicitis?–a comparative study. J Pediatr Surg. (2007) 42:1864–8. 10.1016/j.jpedsurg.2007.07.013 18022437

[7] Beek MA Jansen TS Raats JW Twiss EL Gobardhan PD van Rhede van der Kloot EJ. The utility of peritoneal drains in patients with perforated appendicitis. Springerplus. (2015) 4:371. 10.1186/s40064-015-1154-9 26217548 PMC4512985

[8] Aneiros Castro B Cano I Garcia A Yuste P Ferrero E Gomez A. Abdominal drainage after laparoscopic appendectomy in children: an endless controversy? Scand J Surg. (2018) 107:197–200. 10.1177/1457496918766696 29628008

[9] Song RY Jung K. Drain insertion after appendectomy in children with perforated appendicitis based on a single-center experience. Ann Surg Treat Res. (2015) 88:341–4. 10.4174/astr.2015.88.6.341 26029680 PMC4443266

[10] Ferris M Quan S Kaplan BS Molodecky N Ball CG Chernoff GW. The global incidence of appendicitis: a systematic review of population-based studies. Ann Surg. (2017) 266:237–41. 10.1097/SLA.0000000000002188 28288060

[11] Dandapat MC Panda C. A perforated appendix: should we drain? J Indian Med Assoc. (1992) 90:147–8. 1522303

[12] Fishman SJ Pelosi L Klavon SL O'Rourke EJ. Perforated appendicitis: prospective outcome analysis for 150 children. J Pediatr Surg. (2000) 35:923–6. 10.1053/jpsu.2000.6924 10873036

