# Supplementary material for: Prophylactic Drain Placement in Childhood Perforated Appendicitis: Does Spillage Matter?
Source: Front Pediatr. 2020 Oct 9;8:588109. doi: 10.3389/fped.2020.588109 (PMC7581796; doi:10.3389/fped.2020.588109)
Supplement: Supplementary file 1 [file Table_1.docx]

| **Comorbidity** | **Operative technique** | **Drain** | **Gender** | **Age [y]** | **Antibiotics** |
| --- | --- | --- | --- | --- | --- |
| severe psychomotor developmental disorder | conversion | yes | male | 11,29 | yes |
| Crohn's disease | laparoscopic | no | female | 14,89 | yes |
| severe developmental disorder | laparotomy | yes | male | 17,22 | yes |

**Supplemental Table 1** Patients excluded from the study.
